# Quality-of-life findings from a randomised phase-III study of XELOX *vs* FOLFOX-6 in metastatic colorectal cancer

**DOI:** 10.1038/sj.bjc.6605442

**Published:** 2009-11-17

**Authors:** T Conroy, M Hebbar, J Bennouna, M Ducreux, M Ychou, G Llédo, A Adenis, R Faroux, C Rebischung, L Kockler, J Y Douillard

**Affiliations:** 1 Centre Alexis Vautrin and Nancy University, EA 4360, Vandoeuvre-lès-Nancy 54511, France; 2 Department of Medical Oncology, University Hospital, Lille 59000, France; 3 Department of Medical Oncology, Centre René Gauducheau, Saint-Herblain 44800, France; 4 Digestive Oncology Unit, Gustave Roussy Institute, Villejuif 94805, France; 5 Department of Medical Oncology, Centre Val d’Aurelle, Montpellier 34298, France; 6 Clinique Saint Jean, Lyon 69008, France; 7 Department of Urologic and Digestive Oncology, Centre Oscar Lambret, Lille 59000, France; 8 Department of Gastroenterology, Centre Hospitalier Départemental Les Oudairies, La Roche Sur Yon 85925, France; 9 Department of Oncology and Haematology, University Hospital, Grenoble 38043, France; 10 Laboratoires Roche, Neuilly-sur-Seine 92200, France

**Keywords:** QoL, QLQ-C30, CCSQ-FACIT, colorectal cancer, capecitabine

## Abstract

**Background:**

A phase-III trial showed the non-inferiority of oral capecitabine plus oxaliplatin (XELOX) *vs* 5-fluorouracil/leucovorin plus oxaliplatin (FOLFOX-6) in terms of efficacy in first-line treatment of metastatic colorectal cancer. A secondary objective was to compare the quality of life (QoL) and health-care satisfaction of patients.

**Methods:**

Patients were randomised to receive XELOX (*n*=156) or FOLFOX-6 (*n*=150) for 6 months. Quality of life and satisfaction were assessed by the Quality of Life Questionnaire-C30 (QLQ-C30) and Functional Assessment of Chronic Illness Therapy Chemotherapy Convenience and Satisfaction Questionnaire (FACIT-CCSQ), respectively. Patients completed questionnaires at baseline, at Cycle3 (C3) and Cycle (C6) (XELOX) or at C4 and C8 visits (FOLFOX-6) and at their final visit.

**Results:**

A total of 245 and 225 patients were assessed using QLQ-C30 and FACIT-CCSQ, respectively. The completion rates were >80%. Global QoL scores did not differ significantly between groups during the study. According to FACIT-CCSQ, XELOX seemed more convenient (C3/C4, *P*<0.001; C6/C8, *P*=0.009) and satisfactory to patients (C6/C8, *P*=0.003) than FOLFOX-6. At the final visit, XELOX patients spent fewer days on hospital visits (3.3 *vs* 5.3 days, *P*=0.045) and lost fewer hours of work/daily activities (10.2 *vs* 37.1 h lost, *P*=0.007).

**Conclusion:**

XELOX has a similar QoL profile, but seemed to be more convenient in terms of administration at certain time points and reduced time lost for work or other activities compared with FOLFOX-6.

Colorectal cancer (CRC) is one of the commonest cancers worldwide. It ranks third in terms of incidence (about 1 million new cases in 2002) after lung and breast cancer and fourth in terms of mortality (529 000 deaths in 2002) (Parkin *et al*, 2005). In Europe, CRC is the second most common cause of cancer-related death (203 700 deaths in 2004) after lung cancer (Boyle and Ferlay, 2005). Despite increased awareness about CRC and screening, about half of the patients still present with, or subsequently develop, metastatic disease.

Traditionally, 5-fluorouracil (5-FU) with or without leucovorin (LV) was the only drug used in the palliative treatment of patients with metastatic colorectal carcinoma (mCRC), but this treatment was reported to have a limited impact on survival. Recently, encouraging results have been obtained with capecitabine (Xeloda; F Hoffmann-La Roche Ltd, Basel, Switzerland), an oral fluoropyrimidine (Miwa *et al*, 1998; Schüller *et al*, 2000; Reigner *et al*, 2001). In the first-line treatment of mCRC, capecitabine as single-agent treatment was found to be at least equivalent to bolus 5-FU/LV in terms of time to disease progression and overall survival (OS), with higher response rates (Hoff *et al*, 2001; Van Cutsem *et al*, 2001; Cassidy *et al*, 2002). A series of phase-III studies were subsequently performed, which showed the non-inferiority of a combination of oxaliplatin with capecitabine compared with different 5-FU-based regimens plus oxaliplatin in patients with mCRC (Díaz-Rubio *et al*, 2007; Porschen *et al*, 2007; Cassidy *et al*, 2008; Rothenberg *et al*, 2008). On the basis of these results, capecitabine seemed to be an alternative to intravenous 5-FU.

In addition to efficacy data, patient quality of life (QoL), convenience and satisfaction are assuming increasing importance in the assessment of cancer therapies. Quality of life considerations are crucial to understanding the impact of cancer on the patient, especially when treatments are palliative rather than curative (Payne, 1992). On the basis of the American Society of Clinical Oncology, European Medicines Agency and Food and Drug Administration recommendations, health-related Quality-of-Life (HRQoL) questionnaires should be incorporated as secondary assessment criteria in controlled clinical trials conducted in patients with advanced cancers (ASCO, 1996; Beitz *et al*, 1996; EMEA, 2005). Among the available QoL questionnaires, the Cancer Quality of Life Questionnaire-C30 (QLQ-C30) was developed and validated by the European Organisation for Research and Treatment of Cancer (Fayers and Bottomley, 2002). It has already been used in more than 3000 studies worldwide and is translated and validated in 81 languages (EORTC, 2008). Acceptability of QLQ-C30 is excellent in patients suffering from CRC (Conroy *et al*, 2002). The module ‘Chemotherapy Convenience and Satisfaction Questionnaire’ (CCSQ) of the Functional Assessment of Chronic Illness Therapy (FACIT) Measurement System, a collection of HRQoL questionnaires related to the management of chronic illnesses, measures the health-care satisfaction of patients (Webster *et al*, 2003; Yost *et al*, 2005a).

A randomised, open-label, multicentre, phase-III study was conducted to show the non-inferiority of oxaliplatin plus oral capecitabine (XELOX) *vs* FOLFOX-6 (oxaliplatin plus LV, then intravenous bolus 5-FU, followed by infusional 5-FU) in terms of efficacy in the first-line treatment of mCRC in France. In the per-protocol population, XELOX reached a similar overall response rate, the primary study end point, compared with FOLFOX-6. In both the per-protocol and intention-to-treat (ITT) populations, median progression-free survival (PFS) and median OS were also comparable, providing further support for the non-inferiority of XELOX *vs* FOLFOX-6. While considering safety, a similar proportion of patients discontinued chemotherapy because of adverse events in both treatment groups. This trial showed that XELOX and FOLFOX-6 were similar in terms of efficacy and safety (Ducreux *et al*, 2007). One of the secondary objectives of this phase-III study, which is the focus of this study, was to compare the QoL and health-care satisfaction of patients receiving either XELOX or FOLFOX-6 in the first-line treatment of mCRC, on the basis of the QLQ-C30 and FACIT-CCSQ.

## Materials and methods

### Study design

This was a phase-III prospective, randomised, multicentre, open-label trial. It was designed to show the non-inferiority of XELOX *vs* FOLFOX-6 in terms of efficacy in the first-line treatment of mCRC. Assessment of patients’ QoL and health-care satisfaction, as well as the health economic impact of both treatments, was the secondary objective. Eligible patients were assigned to a treatment group according to a centralised, balanced (1 : 1) and adaptive randomisation procedure. This procedure was based on a minimisation method with centre, Köhne predictive factors (Köhne *et al*, 2002) and previous chemotherapy as stratification factors.

**Figure 1:**
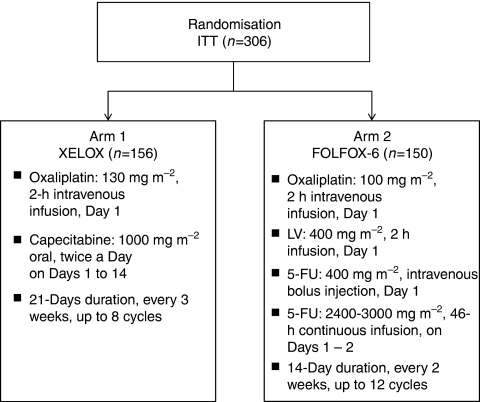
Study treatment schema. 5-FU, 5-fluorouracil; LV, leucovorin.

Two first-line chemotherapy regimens were tested: the XELOX regimen in arm 1 and the FOLFOX-6 regimen in arm 2 (Figure 1). The study comprised a screening visit (baseline) within 14 days before inclusion visit on Day 1 (just before Cycle 1), a treatment period and a follow-up period (including a study visit every 3 months) until the cutoff date, which was fixed at 18 months after the last patient's inclusion. Treatments were continued for 24 weeks (up to 8 cycles with XELOX or 12 cycles with FOLFOX-6) or until disease progression, whichever came first. Study treatment was discontinued in patients experiencing prolonged toxicity (>3 weeks). Dose modifications were made according to previous publications (Cassidy *et al*, 2004, 2006).

Patients were randomised between May 2003 and August 2004 and followed up for 18 months until clinical cutoff in December 2006. The total study duration was 47 months. The study was conducted in France at 33 oncology centres and carried out in accordance with the Declaration of Helsinki and Good Clinical Practice Guidelines. An Independent Ethics Committee approved the protocol. Written informed consent was obtained from all patients participating in the study.

### Patient population

Adult patients (at least 18 years of age) with previously untreated, histologically proven mCRC (at least one measurable target lesion using Response Evaluation Criteria In Solid Tumors) (Therasse *et al*, 2000), an Eastern Cooperative Oncology Group performance status ⩽2 and a life expectancy of >3 months were eligible. Moreover, patients were required to have normal renal function and adequate haematological and hepatic function. Patients with rectal cancer and distant metastases, who had previously received preoperative irradiation on the primary tumour, were eligible if they had assessable non-irradiated metastases. Pregnant or breast-feeding women were excluded. Patients who had received neoadjuvant therapy within the last 6 months containing oxaliplatin, 5-FU or capecitabine, or patients with a history of neuropathy, or uncontrolled congestive heart failure, angina pectoris, hypertension or myocardial infarction within the last 12 months were excluded.

### HRQoL measures

Assessment of HRQoL was based on QLQ-C30 (version 3) (EORTC, 2008) and the CCSQ module from the FACIT scale (Yost *et al*, 2005a). The QLQ-C30 has been previously shown to be a reliable and valid measure of the QoL of cancer patients in multicultural clinical research settings (Aaronson *et al*, 1993). The FACIT-CCSQ convenience items were worded to capture patients’ expectations of chemotherapy and took into account patients’ experience of chemotherapy, satisfaction items and patients’ use of health-care resources within the previous cycle. Both questionnaires were validated in the French language (Conroy *et al*, 2004; Rotonda *et al* 2008; www.facit.org).

**Figure 2:**
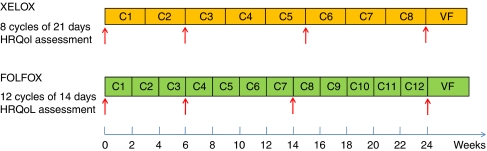
Questionnaires assessment schedule. Each red arrow corresponds to a questionnaire delivery. VF is final visit. The colour reproduction of the figure is available on the html full text version of the paper.

Both QLQ-C30 and FACIT-CCSQ were self-administered by patients enrolled into the study at baseline and at similar time points in both groups during the treatment period. Quality of life assessments were carried out at the same time as tumour imaging assessments and only during the treatment period, that is, at baseline, at Cycle 3 (C3) and Cycle 6 (C6) visits (XELOX) or Cycle 4 (C4) and Cycle 8 (C8) visits (FOLFOX-6) and final visit (Day 169). The timing schedule of QoL and satisfaction assessments is presented in Figure 2.

The QLQ-C30 used was a past week time-framed questionnaire, including 30 items and 15 independent subscores. At each QLQ-C30 assessment, five functional scales (physical, role, cognitive, emotional and social), a global QoL scale and nine symptom/item scales (fatigue, nausea and vomiting, pain, dyspnoea, sleep disturbance, appetite loss, constipation, diarrhoea and financial difficulties) were completed. Each was converted into a scale ranging from 0 to 100. For the QLQ-C30, higher scores represent a better health state for the five functional scales and for the global QoL scale, whereas lower scores represent a better health state for the symptom/item scores. The self-administered FACIT-CCSQ consists of 15 qualitative items relating to the patient's experience of chemotherapy and treatment without time limitations (except one past week time-framed item) and eight items (quantitative criteria) relating to the patient's use of health-care resources during the previous cycle. Most of the qualitative items were organised into three subscales related to chemotherapy convenience, concerns and satisfaction. Each scale and item was transformed into a scale ranging from 0 to 100. The FACIT-CCSQ was translated into the French language according to the FACIT procedure.

### MID

The minimally important difference (MID) is defined as the smallest difference in score in the domain of interest that patients perceive as important, either beneficial or harmful, and that would lead the clinician to consider a change in the patient's management (Guyatt *et al*, 2002; Yost *et al*, 2005b). Considering the QLQ-C30, an MID of more than 10 points from baseline to a subsequent visit could be considered as being clinically significant (Osoba *et al*, 1998). Considering the FACIT-CCSQ, the MID was fixed at 6–9 points for convenience, 7–10 points for concerns and 5–9 points for satisfaction subscales (Yost *et al*, 2005a).

### Statistical methods

The ITT population included all randomised patients meeting the inclusion criteria. Two HRQoL sets were considered:

- QLQ-C30 set: defined as all ITT patients having an assessable QLQ-C30 (<50% of missing responses) at baseline,
- FACIT-CCSQ set: defined as all ITT patients having an assessable FACIT-CCSQ (<50% of missing responses) at baseline.

The number of required patients in the study was based on the demonstration of non-inferiority in terms of efficacy between the XELOX and FOLFOX-6 arms. The total number of patients needed for randomisation in the per-protocol population was defined as 137 persons per group. When considering that 10% of patients could be excluded from the study, an ITT population of (137 × 2)/0.9=304 patients was required. The same number of patients was considered for the secondary objectives, including QoL and satisfaction assessments. Efficacy and safety assessments, as well as health economic results, are presented in detail in separate papers (Perrocheau *et al*, 2009; Ducreux *et al*, 2007).

At each assessment time, the 15 scales of the QLQ-C30 were computed and analysed in the QLQ-C30 set, whereas the items of the FACIT-CCSQ were analysed in the FACIT-CCSQ set. For both questionnaires, missing items were estimated according to their respective scoring manuals when feasible (Fayers *et al*, 2001; www.facit.org). For each item of both questionnaires, the baseline value and values at subsequent visits were provided for each study arm using descriptive statistics. The differences between arms were tested with an analysis of variance test.

Multivariate analyses of OS and PFS were performed using a Cox model with a non-inferiority margin for the hazard ratio fixed at 1.75 and a power of 90%. The analyses included several variables such as age, Köhne score, time interval between CRC diagnosis and metastatic disease, type of cancer, QoL and health-care satisfaction. Differences in QoL and morbidity were analysed using analysis of variance, accounting for differences in survival between groups. Mortality was compared using Kaplan–Meier curves and log–rank statistics. The primary analysis of QoL data was performed using a mixed-models analysis of variances for repeated measures.

The reliability of the multi-item scales of QLQ-C30 and FACIT-CCSQ was assessed using Cronbach *α* coefficient for internal consistency (Cronbach, 1951). A Cronbach *α* coefficient >0.5 was considered as acceptable reliability, whereas a Cronbach *α* coefficient >0.7 was considered as good reliability (Nunally and Berstein, 1994).

## Results

### Study population

A total of 306 patients were randomised: 245 patients (XELOX *n*=126, FOLFOX-6 *n*=119) answered the QLQ-C30 and 225 patients (XELOX *n*=111, FOLFOX-6 *n*=114) answered FACIT-CCSQ. The characteristics at baseline of these patients are presented in Table 1. No significant differences between XELOX and FOLFOX-6 groups regarding socio-demographic data, mCRC characteristics and other baseline characteristics were observed in the HRQoL sets.

### QoL results

#### Completion rate

The completion rate of QoL assessments referred to the number of patients participating at the related visit. Overall, completion rates were satisfactory at the different visits, and were >80% for both questionnaires and for treatment groups.

#### Missing item rate

The missing item rate referred to the number of missing items of data among received forms at the related visit. Overall, the missing item rates for both questionnaires were not significantly different between XELOX and FOLFOX-6 at baseline and at subsequent visits.

#### The European Organisation for Research and Treatment of Cancer QLQ-C30 scores

No relevant differences were observed between the FOLFOX-6 and XELOX arms at baseline and at subsequent visits. Compared with the FOLFOX-6 group, patients had significantly less dyspnoea in the XELOX group (18.0 (13.9; 22.1)_90%_ *vs* 25.4 (20.9; 29.8)_90%_; *P*=0.017), but significantly more sleep disturbances (38.4 (33.3; 43.5)_90%_ *vs* 29.1 (24.4; 33.7)_90%_; *P*=0.036) at baseline. For all subsequent evaluations (C3/C4, C6/C8 and final visit), the QLQ-C30 functional and symptom scores were not significantly different between the XELOX and FOLFOX-6 arms. Results of the QLQ-C30 are summarised in Table 2. Moreover, when focusing on sleep disturbances and dyspnoea items, there were no clinically relevant changes between baseline and final visit for either group, as the changes in scores were <10.

#### FACIT-CCSQ scores

**Figure 3:**
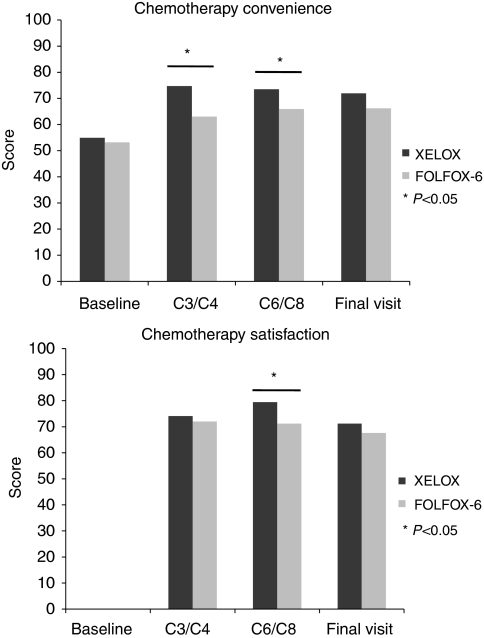
FACIT-CCSQ assessment of chemotherapy convenience and satisfaction in patients receiving either XELOX or FOLFOX-6. CCSQ, Chemotherapy Convenience and Satisfaction Questionnaire; FACIT, Functional Assessment of Chronic Illness Therapy.

No relevant differences between groups were observed at baseline in the FACIT-CCSQ scales. Compared with the FOLFOX-6 group, patients in the XELOX group reported significantly better chemotherapy convenience at C3/C4 (74.7 (71.5; 77.9)_90%_ *vs* 63.0 (59.2; 66.7)_90%_; *P*<0.001) and C6/C8 (73.5 (69.6; 77.3)_90%_ *vs* 65.9 (62.3; 69.4)_90%_; *P*=0.009), as well as better chemotherapy satisfaction at C6/C8 (79.4 (75.3; 83.6)_90%_ *vs* 71.2 (67.3; 75.1)_90%_; *P*=0.003) (Figure 3). At the final visit, XELOX patients spent fewer days on hospital visits (3.3 days (1.5; 5.1)_90%_ *vs* 5.3 days (3.4; 7.1)_90%_; *P*=0.045) and saved more hours of work or usual daily activities (10.2 h lost (3.5; 16.9)_90%_ in the XELOX group *vs* 37.1 h lost (17.4; 56.8)_90%_ in the FOLFOX-6 group, *P*=0.007). No other significant differences between groups were shown for this questionnaire. Results of FACIT-CCSQ are summarised in Table 3.

Moreover, as the MID reached more than 6 points for convenience and more than 5 points for the satisfaction subscales (Yost *et al*, 2005a), the differences observed between the XELOX and FOLFOX-6 groups could be considered as clinically relevant for satisfaction with XELOX at C6/C8, as well as for convenience with XELOX at C3/C4 and C6/C8 visits.

### Multivariate analysis

The results of multivariate analyses showed that all items of the QLQ-C30 had a significant correlation with PFS (except for the cognitive scale, emotional scale and financial difficulties item) and OS (except for the financial difficulties item).

The FACIT-CCSQ items had no correlation with PFS. Only the global quality-of-life score of FACIT-CCSQ had a significant correlation with OS.

### Reliability of scales

All QLQ-C30 multi-item scales, except for the cognitive functional scale at the final visit, showed at least acceptable reliability (data not shown). The Cronbach *α* coefficients for the multi-item scales of FACIT-CCSQ showed good reliability on an average and at least acceptable reliability (data not shown).

## Discussion

This study was the first clinical trial to use both the European Organisation for Research and Treatment of Cancer QLQ-C30 and CCSQ module from FACIT to assess patient QoL and satisfaction with first-line treatment of mCRC. The objective of this study was to evaluate patients’ QoL and health-care satisfaction in the XELOX and FOLFOX-6 groups. Patients in the XELOX group reported significantly better convenience at C3/C4 and C6/C8 visits, as well as better satisfaction at C3/C4, than did patients in the FOLFOX-6 group according to FACIT-CCSQ. Moreover, XELOX patients spent fewer days on hospital visits and saved more hours of work or activity time at the final visit. No differences between groups were shown using QLQ-C30, but the FACIT-CCSQ highlighted that patients seemed more satisfied in the XELOX group than in the FOLFOX-6 group at certain time points. The assessment of internal reliability, based on Cronbach *α* coefficients, revealed a good level of construct validity of both questionnaires.

No clinically relevant differences in QoL between groups were shown using QLQ-C30. This result is not surprising, as XELOX and FOLFOX-6 have similar safety and efficacy profiles (Ducreux *et al*, 2007). Our study showed that the FACIT-CCSQ assessment showed a significant difference between the two treatment regimens in days for hospital visits and hours lost for work or other activities. However, no difference was found between the two regimens in any of the measures for ‘functioning’ in the QLQ-C30 assessment. This could be partly explained by the fact that patients answered the QLQ-C30 just before their hospital visit. Moreover, the assessment related to the previous week when the patient did not receive any chemotherapy. Multivariate analyses showed a significant correlation between most of the QLQ-C30 items and PFS, as well as OS. These results are consistent with those of other studies. Indeed, it has been shown that several QLQ-C30 scales have prognostic value for survival in mCRC (Efficace *et al*, 2006; Efficace *et al*, 2008).

The assessment of QoL and satisfaction of mCRC patients was a secondary objective of this clinical trial. In this context, these data have some limitations. The baseline QLQ-C30 profiles were similar in the XELOX and FOLFOX-6 arms, except for dyspnoea and sleep disturbances. Patients had significantly less dyspnoea and more sleep disturbances in the XELOX group than in the FOLFOX-6 group at baseline, although these differences were not clinically relevant as the differences in scores were less than the MID of 10 points. When considering the changes from baseline to final visit, these between-group differences were no longer evident. Finally, completion rates and item missing rates at different time points were not different between study arms, providing further support for the comparability of treatment groups. The decrease in the number of patients at subsequent visits was similar in both groups and could be partly explained by the patient's tumour status.

Despite these limitations, the results are consistent with other QoL and patient preference studies of oral fluoropyrimidines *vs* intravenous 5-FU/LV in CRC. First, a series of studies have shown the preference of patients for oral treatment (Borner *et al*, 2002; Kopec *et al*, 2007). For example, Kopec *et al* (2007) showed similar results in patients with stage II/III carcinoma of the colon who received oral uracil/ftorafur (UFT) plus LV or standard intravenous 5-FU/LV as adjuvant chemotherapy. Health-related Quality-of-Life was measured with the Functional Assessment of Cancer Therapy-Colorectal (FACT-C), the Short Form-36 Vitality Scale and a Quality of Life Rating Scale. Patients perceived adjuvant treatment with UFT/LV as being more convenient than standard IV treatment with 5-FU/LV. Both regimens were well tolerated and did not differ in their impact on HRQoL. Moreover, two recent studies underlined the preference of patients for capecitabine over 5-FU. Pelusi (2006) confirmed that the oral approach was preferred by patients because of its convenience (fewer medical office visits, no intravenous access required) and by clinicians because it eliminated the risk of complications, such as infection and clotting associated with venous access devices and infusion pumps. In another study conducted by Twelves *et al* (2006), 97 patients with previously untreated advanced or mCRC were randomised to receive capecitabine, followed by intravenous 5-FU/LV (Mayo Clinic, in-patient de Gramont or outpatient modified de Gramont regimens), or intravenous 5-FU/LV followed by capecitabine. Quality of life was assessed with the FACT-C questionnaire. The results confirmed that the majority of patients with mCRC preferred oral therapy.

Health economic results based on the current clinical trial further showed that XELOX significantly decreased the direct treatment costs of mCRC patients, as well as hospital resource consumption, in comparison with FOLFOX-6 (Perrocheau *et al*, 2009). Considering clinical and economic impacts, the XELOX regimen seems to be a relevant alternative to FOLFOX-6 in the first-line treatment of mCRC.

## Conclusion

This study was the first clinical trial to evaluate QoL and health-care satisfaction in patients receiving XELOX in the first-line treatment of mCRC. XELOX has a similar QoL profile, but seems to be more convenient in terms of administration at certain time points and reduced time lost for work or other activities compared with FOLFOX-6. Therefore, capecitabine, used in the XELOX regimen, clearly represents an effective and well-tolerated oral alternative to intravenous 5-FU/LV.

## Figures and Tables

**Table 1:** Baseline demographic characteristics of patients

		**EORTC QLQ-C30**			**FACIT-CCSQ**		
		**XELOX group *N*=126**	**FOLFOX-6 group *N*=119**	***P*-value**	**XELOX group *N*=111**	**FOLFOX-6 group *N*=114**	***P*-value**
Age (years)	*n*_missing_	0	0	0.59	0	0	0.47
	Mean (s.d.)	65.8 (10.0)	65.6 (8.9)		66.0 (9.8)	65.4 (9.4)	
*Gender*	*n*_missing_	0	0	0.20	0	0	0.10
Male	*n* (%)	81 (64.3)	67 (56.3)		74 (66.7)	64 (56.1)	
Female	*n* (%)	45 (35.7)	52 (43.7)		37 (33.3)	50 (43.9)	
BMI (kg m^−2^)	*n*_missing_	3	4	0.27	2	4	0.57
	Mean (s.d.)	24.6 (4.2)	25.3 (4.9)		24.5 (4.1)	25.1 (5.0)	
*Localisation in France^a^*	*n*_missing_	0	0	0.08	0	0	0.06
Paris	*n* (%)	9 (7.1)	21 (17.6)		9 (8.1)	22 (19.3)	
Northwest	*n* (%)	48 (38.1)	37 (31.1)		34 (30.6)	25 (21.9)	
Northeast	*n* (%)	33 (26.2)	31 (26.1)		31 (27.9)	31 (27.2)	
Southeast	*n* (%)	25 (19.8)	25 (21.0)		23 (20.7)	28 (24.6)	
Southwest	*n* (%)	11 (8.7)	5 (4.2)		14 (12.6)	8 (7.0)	
*ECOG PS*	*n*_missing_	0	0	0.89	0	0	0.76
0–1	*n* (%)	116 (92.1)	109 (91.6)		101 (91.0)	105 (92.1)	
2	*n* (%)	10 (7.9)	10 (8.4)		10 (9.0)	9 (7.9)	
*Tumour primary site*	*n*_missing_	0	0	0.43	0	0	0.54
Colon	*n* (%)	76 (60.3)	75 (63.0)		66 (59.5)	69 (60.5)	
Colorectum	*n* (%)	28 (22.2)	30 (25.2)		26 (23.4)	31 (27.2)	
Rectum	*n* (%)	22 (17.5)	14 (11.8)		19 (17.1)	14 (12.3)	

Abbreviations: BMI=body mass index; CCSQ=Chemotherapy Convenience and Satisfaction Questionnaire; ECOG PS=Eastern Cooperative Oncology Group performance status; EORTC=European Organisation for Research and Treatment of Cancer; FACIT=Functional Assessment of Chronic Illness Therapy; *N*=number of exposed patients; QLQ-C30=Quality of Life Questionnaire-C30.

a These five French areas correspond to the areas defined by the five area codes (01, 02, 03, 04 and 05).

**Table 2:** EORTC QLQ-C30 questionnaire assessment in the QLQ-C30 set (*N*=245)

	**XELOX group, *n*=126**			**FOLFOX-6 group, *n*=119**			
**EORTC QLQ-C30**	***n***	**MV (%)**	**Mean (s.d.)**	***n***	**MV (%)**	**Mean (s.d.)**	*P-value
*Physical functioning (%)*							
Baseline	126	0.0	80.7 (20.2)	118	0.8	79.1 (21.9)	0.952
C3/C4	98	0.0	80.5 (22.0)	96	1.0	78.9 (19.0)	0.225
C6/C8	78	0.0	80.0 (18.5)	72	2.7	79.5 (18.6)	0.645
Final visit	63	0.0	75.9 (22.0)	65	0.0	74.9 (21.0)	0.631
							
*Role functioning (%)*							
Baseline	124	1.6	71.2 (31.8)	116	2.5	68.2 (32.3)	0.484
C3/C4	98	0.0	73.8 (28.7)	96	1.0	67.4 (30.6)	0.121
C6/C8	78	0.0	74.8 (28.9)	73	1.4	70.5 (27.0)	0.198
Final visit	62	1.6	69.9 (32.1)	65	0.0	65.1 (29.4)	0.206
							
*Cognitive functioning (%)*							
Baseline	123	2.4	83.7 (20.5)	118	0.8	83.2 (22.2)	0.961
C3/C4	98	0.0	85.0 (19.9)	96	1.0	82.3 (20.5)	0.247
C6/C8	78	0.0	79.3 (23.1)	74	0.0	82.0 (19.3)	0.641
Final visit	63	0.0	78.6 (19.3)	64	1.5	77.7 (19.5)	0.631
							
*Emotional functioning (%)*							
Baseline	124	1.6	68.8 (25.2)	117	1.7	68.0 (24.5)	0.715
C3/C4	98	0.0	78.6 (20.2)	96	1.0	75.2 (22.0)	0.333
C6/C8	78	0.0	79.2 (20.4)	74	0.0	75.6 (21.9)	0.285
Final visit	62	1.6	72.7 (23.8)	64	1.5	69.7 (24.2)	0.438
							
*Social functioning (%)*							
Baseline	123	2.4	77.2 (30.1)	116	2.5	76.1 (27.6)	0.461
C3/C4	98	0.0	79.3 (24.8)	96	1.0	72.9 (26.2)	0.058
C6/C8	78	0.0	78.8 (25.4)	74	0.0	78.2 (25.3)	0.924
Final visit	63	0.0	73.5 (28.7)	64	1.5	71.6 (27.0)	0.539
							
*Global quality of life scale (%)*							
Baseline	123	2.4	62.4 (22.8)	116	2.5	59.8 (20.9)	0.372
C3/C4	98	0.0	66.5 (22.2)	96	1.0	62.2 (18.0)	0.050
C6/C8	78	0.0	64.5 (21.7)	73	1.4	62.7 (19.0)	0.349
Final visit	63	0.0	58.1 (22.8)	63	3.1	60.4 (19.1)	0.449
							
*Fatigue (%)*							
Baseline	125	0.8	40.3 (29.1)	117	1.7	38.4 (27.6)	0.626
C3/C4	98	0.0	36.9 (24.9)	97	0.0	40.3 (26.9)	0.510
C6/C8	78	0.0	38.1 (27.5)	73	1.4	40.3 (26.9)	0.536
Final visit	63	0.0	45.1 (27.2)	65	0.0	41.5 (25.9)	0.475
							
*Nausea and vomiting (%)*							
Baseline	125	0.8	6.0 (14.9)	118	0.8	8.2 (19.5)	0.395
C3/C4	98	0.0	11.9 (19.6)	97	0.0	12.2 (22.2)	0.738
C6/C8	78	0.0	10.5 (17.8)	73	1.4	13.0 (21.2)	0.687
Final visit	63	0.0	10.3 (18.3)	65	0.0	10.3 (17.6)	0.991
							
*Pain (%)*							
Baseline	126	0.0	23.3 (27.8)	118	0.8	24.0 (27.6)	0.707
C3/C4	98	0.0	19.0 (24.5)	97	0.0	18.7 (23.0)	0.949
C6/C8	78	0.0	20.9 (28.9)	74	0.0	17.3 (20.0)	0.908
Final visit	63	0.0	31.2 (33.5)	65	0.0	25.4 (26.4)	0.512
							
*Dyspnoea (%)*							
Baseline	123	1.6	18.0 (27.3)	117	1.7	25.4 (28.9)	**0.017**
C3/C4	97	1.0	18.6 (25.9)	95	2.1	18.6 (24.7)	0.890
C6/C8	77	1.3	18.6 (25.6)	73	1.4	19.2 (24.8)	0.774
Final visit	62	1.6	21.0 (27.1)	65	0.0	20.0 (24.9)	0.974
							
*Insomnia (%)*							
Baseline	125	0.8	38.4 (34.1)	117	1.7	29.1 (30.2)	**0.036**
C3/C4	98	0.0	26.5 (31.4)	97	0.0	25.4 (29.6)	0.916
C6/C8	78	0.0	25.6 (31.7)	72	2.7	20.4 (26.6)	0.391
Final visit	63	0.0	28.6 (33.3)	65	0.0	24.6 (27.2)	0.725
							
*Appetite loss (%)*							
Baseline	125	0.8	24.3 (32.9)	116	2.5	26.1 (33.4)	0.550
C3/C4	98	0.0	21.4 (29.2)	97	0.0	24.4 (34.2)	0.798
C6/C8	78	0.0	23.5 (30.4)	73	1.4	19.4 (28.4)	0.424
Final visit	62	1.6	31.2 (36.1)	63	3.1	20.6 (30.8)	0.106
							
*Constipation (%)*							
Baseline	124	1.6	17.2 (29.6)	117	1.7	18.5 (26.8)	0.312
C3/C4	95	3.1	13.0 (21.4)	95	2.1	21.1 (28.8)	0.065
C6/C8	77	1.3	19.0 (27.3)	73	1.4	14.6 (24.8)	0.286
Final visit	61	3.2	22.4 (31.5)	64	1.6	21.4 (28.1)	0.860
							
*Diarrhoea (%)*							
Baseline	124	1.6	16.7 (26.0)	116	2.5	15.8 (26.2)	0.700
C3/C4	98	0.0	21.1 (28.1)	96	1.0	21.2 (30.3)	0.800
C6/C8	77	1.3	21.2 (31.5)	74	0.0	16.2 (25.4)	0.506
Final visit	63	0.0	15.9 (27.3)	64	1.6	7.8 (18.5)	0.072
							
*Financial difficulties (%)*							
Baseline	123	2.4	8.9 (23.4)	114	4.2	10.8 (24.5)	0.515
C3/C4	98	0.0	7.1 (19.3)	95	2.1	9.1 (20.9)	0.477
C6/C8	78	0.0	8.5 (21.8)	74	0.0	11.3 (24.2)	0.289
Final visit	63	0.0	10.1 (22.1)	64	1.6	12.0 (22.5)	0.479

Abbreviations: C3, C4, C6, C8=Cycles 3, 4, 6 and 8; EORTC=European Organisation for Research and Treatment of Cancer; %MV=percentage of missing values; QLQ-C30=Quality of Life Questionnaire-C30.

^*^P value: Student's *t*-test or Wilcoxon test for independent samples. Bold values indicate *P*<0.05.

**Table 3:** FACIT-CCSQ questionnaire assessment in the FACIT-CCSQ set (*N*=225)

	**XELOX group, *n*=111**			**FOLFOX-6 group, *n*=114**			
**FACIT-CCSQ**	***n***	**MV (%)**	**Mean (s.d.)**	***n***	**MV (%)**	**Mean (s.d.)**	*P-value
*Chemotherapy convenience*							
Baseline	111	0.0	54.9 (23.3)	112	1.8	53.2 (19.1)	0.471
C3/C4	93	0.0	74.7 (18.5)	88	0.0	63.0 (21.0)	**<0.001**
C6/C8	65	1.5	73.5 (18.6)	72	0.0	65.9 (18.2)	**0.009**
Final visit	61	0.0	71.9 (19.2)	63	0.0	66.2 (18.0)	0.081
							
*Chemotherapy concerns*							
Baseline	111	0.0	61.4 (19.3)	113	0.9	58.9 (19.8)	0.492
C3/C4	93	0.0	76.3 (19.6)	88	0.0	72.7 (20.6)	0.186
C6/C8	66	0.0	75.3 (19.8)	72	0.0	73.9 (17.7)	0.454
Final visit	61	0.0	71.6 (19.7)	62	1.6	67.6 (19.9)	0.289
							
*Chemotherapy satisfaction*							
Baseline	—	—	—	—	—	—	—
C3/C4	84	10.7	74.1 (21.9)	79	11.4	72.0 (20.0)	0.311
C6/C8	63	4.8	79.4 (19.7)	66	9.1	71.2 (18.9)	**0.003**
Final visit	52	17.3	71.2 (20.5)	55	14.5	67.6 (20.2)	0.296
							
*I worry that my chemotherapy will not be effective (%)*							
Baseline	108	2.7	74.8 (29.3)	110	3.6	77.3 (25.7)	0.707
Final visit	60	1.6	73.8 (30.3)	60	5.0	76.7 (26.4)	0.751
							
*My chemotherapy schedule is stressful to my family (%)*							
Baseline	110	0.9	54.5 (29.6)	114	0.0	57.0 (28.3)	0.571
Final visit	61	0.0	69.3 (30.1)	61	3.3	69.3 (25.2)	0.703
							
*Within past week*							
*I am content with the quality of my life right now (%)*							
Baseline	107	3.7	52.3 (28.0)	109	4.6	50.7 (26.2)	0.553
Final visit	58	5.2	46.1 (28.4)	59	6.8	45.3 (23.0)	0.711
							
*Within past cycle*							
*Hospital visits*							
Final visit	39	36.1	1.9 (4.5)	40	36.5	2.1 (4.6)	0.974
							
*Emergency room admissions*							
Final visit	31	49.2	0.0 (0.0)	33	47.6	0.0 (0.0)	1.000
							
*Physician visits*							
Final visit	38	60.4	0.6 (1.7)	37	41.3	0.3 (1.0)	0.295
							
*Number of days for a usual hospital visit*							
Final visit	31	49.2	3.3 (5.9)	27	57.1	5.3 (5.6)	**0.045**
							
*Number of hours for a usual emergency room admission*							
Final visit	22	63.9	0.2 (0.9)	17	73.0	0.5 (1.5)	0.426
							
*Number of hours for a usual physician visit within past cycle?*							
Final visit	27	55.7	0.6 (1.0)	26	58.7	1.4 (5.9)	0.407
							
*How many hours have you lost for your work or usual daily activities?*							
Final visit	33	45.9	10.2 (22.6)	34	46.0	37.1 (67.9)	**0.007**
							
*How many hours have your friends or your family lost for their work or usual daily activities?*							
Final visit	36	41.0	3.1 (4.6)	32	49.2	20.0 (53.0)	0.059

Abbreviations: C3, C4, C6, C8=Cycles 3, 4, 6 and 8; CCSQ=Chemotherapy Convenience and Satisfaction Questionnaire; FACIT=Functional Assessment of Chronic Illness Therapy; %MV=percentage of missing values.

^*^P-value: Student's *t*-test or Wilcoxon test for independent samples. Bold values indicate *P*<0.05.
